# Homicide-Suicides in Pakistan: An analysis of Newspaper reports of two years

**DOI:** 10.12669/pjms.40.10.9812

**Published:** 2024-11

**Authors:** Nazish Imran, Maryam Ayub, Imran Ijaz Haider, Bariah Rafiq, Sania Mumtaz Tahir, Sadiq Naveed, Murad Moosa Khan

**Affiliations:** 1 Nazish Imran, Department of Child and Family Psychiatry, King Edward Medical University, Lahore, Punjab, Pakistan; 2 Maryam Ayub, Academic Department of Psychiatry and Behavioral Sciences, King Edward Medical University, Lahore, Punjab, Pakistan; 3 Imran Ijaz Haider, Ijaz Clinic/ Connections Psychiatric Services Lahore, Lahore, Punjab, Pakistan; 4 Bariah Rafiq, Academic Department of Psychiatry and Behavioral Sciences, King Edward Medical University, Lahore, Punjab, Pakistan; 5 Sania Mumtaz Tahir, Academic Department of Psychiatry and Behavioral Sciences, King Edward Medical University, Lahore, Punjab, Pakistan; 6 Sadiq Naveed, Frank H. Netter School of Medicine, Quinnipiac University, CT. United States of America; 7 Murad Moosa Khan, Department of Psychiatry, Aga Khan University, Karachi, Sindh, Pakistan

**Keywords:** Homicide-Suicide, Murder–suicide, Filicide, Pakistan

## Abstract

**Background and Objective:**

Our understanding of homicide-suicide (H-S), a rare yet tragic event, is sparse. While the phenomenon has been studied in the West for many years, only limited literature is available from Asia and none to our knowledge from Pakistan. There is evidence of complexity of the interaction between cultural, societal, and psychological mechanisms underlying this phenomenon; therefore, research findings from the Western countries cannot be directly applied in non-Western societies. Our objective was to study homicide-suicides in Pakistan, describe the characteristics of offenders and victims, determine the types of H-S, and examine possible motives and any events prior to the offense.

**Methods:**

In the absence of any official data on homicide-suicides, we used newspaper surveillance approach of four most widely circulated Pakistani newspapers (one Urdu and three English Daily) for two years (1^st^ January 2019 to 31^st^ December 2020). Each case was categorized using the modified Marzuk et al., Tardiff, and Hirsch’s classification of homicide-suicides. Descriptive statistics were used to analyze the data that was then compared with published literature.

**Results:**

There were 114 H-S incidents with 198 victims of homicide during the study period, reported in the newspapers. Familial H-S particularly filicide-suicide were predominant, followed by spousal/ consortial H-S. Forty-one (36%) H-S involved multiple victims. The perpetrators across all categories of H-S were predominantly male (67%); the victims were predominantly women and children. Firearms were used in most incidents. The primary motive for the majority of H-S cases was familial, financial, and social stressors.

**Conclusions:**

The study highlights several unique patterns (predominance of familicide, multiple victims including high proportion of children) and a series of vulnerabilities (incidents related mostly to familial/ financial and social stressors) that overlap each other and ultimately lead to this tragic end. There is need to increase our understanding and develop effective evidence-based prevention strategies for H-S in Pakistan. It is also very important to have a national surveillance network and national violent death reporting system in the country for studying H-S cases, and for evaluating the impact of prevention programs.

## INTRODUCTION

Homicide-suicide (H-S), also known as dyadic death, is a rare but significant phenomenon involving the killing of one or more individuals followed by the suicide of the perpetrator.^1^ Despite its rarity, H-S events have a profound and lasting impact on individuals, families, and communities, often garnering substantial media attention.^2^ Unlike typical criminal cases, there’s no standardized legal description for H-S. It’s considered a unique epidemiological phenomenon with similarities to both homicide and suicide. While research traditionally focuses on one or the other, H-S warrants attention as a distinct public health issue.^3^

Despite H-S being a universal phenomenon, studies on H–S are scarce, and majority are published from western countries. A large-scale systematic review of 49 homicide-suicides revealed that most of homicide-suicide occurs in a family context.^4^ The perpetrators were usually men, while the victims were usually females.^4^ The official homicide-suicide prevalence rate ranges from 0.27 to 0.38/100,000 inhabitants in the United States,^5^ 0.05 in England & Wales,^6^ 0.04 in Italy,^7^ 0.2 in Finland^2^ and 0.89 in South Africa.^8^ Data from Asia seems to be particularly lacking with one study from Japan (0.44/100000 people) found during literature review in 2001^9^ and a study from Hong Kong in 2003, noted 0.09 (H-S events) per 100,000 persons per year.^10^

In India, during the five years period from 2000 to 2004, the annual incidence of homicide–suicide was about 0.06 per 100,000 in a study done in Gujarat.^11^ The dearth of research on homicide–suicides in Asian countries are unfortunate for various reasons. First, relatively little is currently known about the epidemiology of homicide–suicides for a large proportion of the global population. Second, there is evidence of complexity of the interaction between cultural, societal, and psychological mechanisms underlying this phenomenon; therefore, research findings from the Western countries cannot be directly applied to explain homicide–suicides in non-Western societies.^11^ Third, until there is an in depth understanding of H-S as a phenomenon through research, it will be difficult to develop and implement appropriate preventive interventions to curb these tragic incidents.^12^

Pakistan is the fifth most populous country globally and second-most populous country in South-East Asia, with an estimated population of 207 million. In Pakistan, homicide-suicide incidents are regularly reported in the media but so far there is absence of any studies related to the phenomenon. There are no official data on suicides in Pakistan nor does it report any suicide data to World Health Organization. Newspapers surveillance approach is not new or unusual, and though limited in scope and the information they contain, in settings where there is scarcity of official data, particularly in developing countries, has proven to be an acceptable and useful source of information.^14^,^15^ Considering several recent reports of H-S in the media in Pakistan, the current study aims to highlight the phenomena, by examining all homicide-suicide incidents reported over two years in multiple national newspapers of Pakistan and compare the results with studies available from other countries.

## METHODS

After Institutional Review Board approval from King Edward Medical University (Ref. No.: 615/RC/KEMU; September 7, 2021), data for the present study were extracted from reports on homicide-suicide deaths for duration of two years from 1^st^ January 2019 to 31^st^ December 2020 from four Pakistani newspapers. These included Daily Jang (Urdu Language) and Daily Dawn, The News & the Nation (three English language newspapers). All these newspapers are among top five overall circulated newspapers in the country.^16^ All city editions were reviewed, which include five city editions of The Nation, three city editions of Tribune, four city editions of Dawn and five city editions of Daily Jang. Homicide-suicide for this study was operationalized as a homicide followed by suicide of perpetrator up to one week later.^1^ This definition required the presence of fatal outcomes in both the homicidal and suicidal acts. Cases where homicide-suicide was attempted but one or both parties did not die or which were not H-S reports from Pakistan were excluded. Authors came across argument about “victims” in H-S discussion to refer to *everyone* who died, including the person who died by suicide. But in line with many previous studies, we operationalized the “victim” in current study as persons in H-S incidents, who were murdered.^4^,^10^

Four authors extracted the data and entered it in a spreadsheet designed specifically for this purpose, coordinated by one of senior authors having command over both languages, who also rechecked and edited data to avoid any double reporting of H-S cases. Each case was categorized using the modified Marzuk et al, Tardiff, and Hirsch’s [MTH] (1992) classification of homicide-suicides^1^ as this system has been used by other authors in analyses of homicide-suicide acts.^17^ Marzuk et al. typology is principally based on the type of victim-offender relationship and by class of the primary motive underlying the offence. It is useful in providing clinicians with a simple mental construct for assessing the risk of this type of violence. The main types of homicide-suicide based on victim-offender relationship described by Marzuk et al. (1992) are spousal (uxoricide) /consortial couples; filicide-suicide in which the homicide victim (or victims) was a child 0–16 years old; Familicide-suicide (an overlap between the Uxoricide & Filicide categories: the killing of both spouse and children); *killing* of other family members followed by suicide; and non-familial homicide-suicide in which the homicide victim (or victims) was not a family member.^1^ Classes by motives include Amorous jealousy, mercy killing, Altruistic suicides, family financial or social stressors, retaliation, others and unspecified. Descriptive statistics were used to analyze the data extracted from the newspaper reports, using SPSS 26.

### Ethical approval:

All procedures contributing to this work comply with the ethical standards of the relevant national and institutional committees on human experimentation and with the Helsinki Declaration of 1975, as revised in 2008. Ethical approval was obtained from the Institutional Review Board of King Edward Medical University (Ref. No.: 615/RC/KEMU; dated: September 7, 2021).

### Patient’s consent:

Not applicable.

## RESULTS

### Demographic characteristics:

During the study period, there were a total 114 (69 in 2019 and 45 in 2020) homicide-suicides reported in the four selected Pakistani newspapers. Table-I Types of Homicide-Suicide are described in Table-II, according to Marzuk et al., Tardiff and Hirsch (MTH) Typology (1992) with Familial H-S observed to be most prevalent type. Among Familicide subtypes, 44 cases were of Filicide-suicide.

**Table-I:** Characteristics of homicide-suicide Total (N=114).

Variables	N=114 (n)	%
***Demographic Characteristics***		
***Province***		
Punjab	77	67.5
Sindh	27	23.7
KPK	6	5.3
Baluchistan	4	3.5
***Area***		
Urban	67	58.8
Rural	33	28.9
Not Available	14	12.3
***Location***		
Inside home	55	48.2
Outside home	18	15.8
Canal	13	11.4
Hospital	1	0.9
Roadside in car	1	0.9
Train track	1	0.9
Well	1	0.9
Field	1	0.9
Not Available	41	36
***Offence Characteristics***		
***Suicide occurred < 24 hours after homicide.***		
Yes	101	88.6
No	0	0
Not Available	13	11.4
***Homicide and suicide occurred in shared place***		
Yes	84	73.7
No	0	0
Not Available	30	26.3
***Number of Homicide Victims in one incident***		
One	73	64.0
Two	19	16.7
Three	11	9.6
Four	9	7.9
Five	1	0.9
Six	1	0.9
***Method of homicide***		
Firearm	53	46.5
Drowning	18	15.8
Poisoning	16	14.0
Sharp Weapon	15	13.2
Fire/smoke	2	1.7
Strangulation/Hanging/Asphyxia	2	1.7
Hit and run	1	0.9
Others	3	2.6
Not Available	4	3.5
***Method of suicide***		
Firearm	53	46.5
Poisoning	16	14.0
Drowning	16	14.0
Strangulation/Hanging/Asphyxia	8	7.0
Sharp Weapon	7	6.2
Fire/smoke	3	2.6
Not Available	11	9.6
***Precipitating Motive/ Class based on Marzuk, Tardiff and Hirsch (MTH), (1992) Typology***		
Amorous Jealousy	10	8.8
Mercy Killing	0	0.0
Altruistic or extended suicide	5	4.4
Familial/ financial and social stressors	60	52.6
Retaliation	3	2.6
Others	6	5.3
Unspecified	30	26.3

**Table-II:** Types of homicide-suicide according to Marzuk, Tardiff and Hirsch, (1992) (MTH) Typology.

Type of Homicide-Suicide	N=114 n	%
Spousal/ Consortial	35	30.7
Uxoricide-Suicide (The killing of spouse and then subsequently him/herself)	25	21.9
Consortial	10	8.8
Familial		
Filicide-suicide *(The killing of one’s children and then oneself.)*	71	62.2
Familicide-suicide	44	38.6
(An overlap between the Uxoricide & Filicide categories: the killing of both spouse and children).	11	9.6
Killing of other family members followed by suicide	16	14
Extra familial (*Victims outside the familial sphere)*	8	7.0

### Characteristics of Homicide-Suicide Perpetrators:

The age of perpetrator was reported only in 43 reports and ranged from 17 to 60 years with mean age of 35.6 (s. d=12.43) years. Majority of offenders were male (76, 66.7%) and married (76%). In 38 of the 39 incidents, where women were the perpetrator, victims were her children (Filicide-Suicide). Perpetrator in these incidents were married (31), separated (1), widowed (1) while marital status was unclear from the reports in five incidents. Mental illness was mentioned in only five (4%) cases and substance abuse in only 1% of the reports.

### Characteristics of Homicide-Suicide Victims:

There were a total of 198 victims of 114 incidents. Table-III gives further details about H-S victims.Table-III

**Table-III:** Characteristics of homicide-suicide victim (N=198).

Victim Characteristics	n	%
***Age Groups***		
0-5	61	30.81
5-10	14	7.07
10-15	4	2.02
15-20	10	5.05
20-30	19	9.60
30-40	5	2.53
40-50	6	3.03
50-60	2	1.01
>60	1	0.50
Not Available	76	38.4
***Gender***		
Female	138	69.7
Male	59	29.8
One full term fetus (Gender not mentioned)	1	0.50
***Marital Status***		
Married	65	32.8
Divorced	1	0.5
Not Available	132	66.7
***Relationship of victim with perpetrator***		
***Family***	131	66.1
Child	25	12.6
Spouse	13	6.6
Sibling	4	2.0
Parent	11	5.6
Others		
***Non-family***		
Love interest	10	5.0
Friend	1	0.5
Acquaintance	3	1.5

## DISCUSSION

To the best of our knowledge this is the first study on homicide–suicide incidents from Pakistan. We found 114 homicides- suicides reports in selected newspapers in two years, which is a cause of concern. Also, in the absence of official H-S statistics and the methodology we used (newspaper surveillance), this is most likely an underestimate. In contrast a three years case series in England recorded sixty incidents, while a similar study from France over a six-year period showed 10 cases.^18^,^19^ A study from India looking at National Crime Bureau records for the year 2014 identified seventy-two cases. Whether H-S is more common in South Asia compared to West is difficult to say and only systematic data collection and surveillance would confirm this. Our data were similar in some respects to other studies that showed a close temporal proximity between homicide and suicide and majority of victims (92%) were family members.^19^,^20^ However, in contrast to other studies, where most victims were spousal/consortial, involving a man killing his wife, girlfriend, ex-wife, or ex-girlfriend,^4^,^19^,^20^ in our study we observed highest percentage (62%) of familial H-S with predominance (38.6%) of filicide-suicide incidents (killing of one’s children and then oneself). More than one third of all victims in our study were either intimate partners or children. Malphurs et al. and Cohen et al. (2002) in the newspaper analysis of homicide suicide in USA found that 70.5 percent of all H-S were spousal/consortial, 6.5% were familicides and 8.7% were extrafamilial.^21^

Our study found multiple victims in 41 (36%) H-S incidents. Flynn et al. (2016) and Saint-Martin et al. observed more than one victim in only six and one. H-S incidents respectively, while multiple victims (148) were recorded in 72 cases from India.^18^,^19^ A higher number of multiple victims may be explained by overall higher incidents of filicide suicide and familicide-suicide in our study.

Homicide-suicide (H-S) exhibits a diverse range of motivational factors, reflecting the complex interplay of psychological, situational, and demographic variables. While psychiatric illnesses play a role, H-S typically results from a combination of various bio-psycho-social and environmental factors. Contrary to some findings which state that HS occurs mostly in intimate relationship conflicts,^4^ our study reveals that family, financial, and social stressors are predominant in H-S cases. In some previous studies, it has also been shown that interpersonal dynamics preceding H-S often involve poverty, debts, and financial issues,^4^,^22^,^23^ with “altruistic murder” observed in cases where individuals seek to spare loved ones from suffering alone or aim to “leave together.”^24^ Firearms are commonly chosen as the method of carrying out H-S acts, consistent with findings from other studies.^4^,^7^,^12^,^19^,^21^

Mental illness appears to play an important role in HS phenomena.^4^,^7^ Depression was the most frequently reported disorder (39%) in 20 studies that assessed such disorders, followed by substance abuse and psychosis.^7^ Correlatively, the perpetrators’ clinical profile appears to be marked with recurrent self-harm, suicidal thoughts, and prior suicide attempts.^4^ The information available from our data suggested evidence of psychiatric illness and substance abuse in only six incidents but this may be a reflection of lack of information in the news reports. One of the most consistent findings in literature is that men carried out most homicides-suicides and victims were usually female and/or children.^4^,^7^ According to our study, 67% of perpetrators were males, while 70% of victims were females. Only 8% of victims were nonfamily similar to a systemic review of 30 studies, where it was seen that homicide followed by suicide usually occurs between family members.^7^ Thus, the closer the perpetrator and the victim(s) are to each other the greater is the risk of HS.^18^ Age of perpetrator was mentioned in only 43 incidents in our study with age range of 17-60 years. Most of the previous studies reported perpetrators’ to be 40-50 years of age.^20^ As the information provided in many newspaper reports was incomplete, it was not possible to analyze the data regarding age, education level and social class of subjects adequately. This limitation needs to be seen in context of the overall paucity of information about homicides and suicides in Pakistan.

The profile of victims that emerges from our study reveals a high proportion of children in the HS incidents, with almost 40% of victims under the age of 15 years. This is in contrast to the findings in studies from the West where Saint-Martin et al. in their study noted no case involving children during the study time of six years in Tours, France^19^ but in line with study by Subba Reddy et al. who observed comparatively high percentages of victims in 0-15 years age group in Hyderabad, India.^23^ A study analyzing data from 46 US states between 2003 and 2013 found that interpersonal stressors and criminal history increase the likelihood of homicide-suicide compared to suicide. Physical and mental health stressors decrease this likelihood. The study suggests that preventing homicide-suicide may be better addressed through violence prevention screening rather than traditional suicide prevention programs.^25^

### Limitations:

The study acknowledges the challenges of relying on retrospective newspaper reports and non-research documents to understand homicide-suicide (HS) events, especially given the limited data availability due to the deceased nature of those involved and the scarcity of such incidents. The main limitation lies in the source of data, which may underestimate the actual frequency of HS incidents, emphasizing the need for systematic data collection through registries and police records. Missing data further hampers statistical analysis, limiting insights into factors such as age, socioeconomic status, and mental health history. Reporting biases and stigma-related underreporting add to these limitations, hindering comparisons with studies from other countries. Despite these challenges, the study contributes to identifying characteristics and risk factors associated with HS phenomena in Pakistan, highlighting patterns and vulnerabilities that lead to such tragic outcomes. It underscores the importance of accurate epidemiological understanding to develop preventive strategies.

## CONCLUSION

Future research is suggested to highlight psychopathological factors associated with HS, employing psychological autopsy methods to gain insights into psychiatric illnesses and motives behind such events, ultimately aiding in the development of preventive measures.

It is very important to have a national surveillance network and national violent death reporting system in the country for studying H-S cases, and for evaluating the impact of prevention programs. Both distal (such as poverty) and proximal factors (e.g., easy availability of firearms) need to be included in a prevention strategy. Based on the points highlighted in this study, policymakers and providers can devise further interventions to address these rare yet extremely tragic incidents.

### Authors’ contribution:

**NI, IIH, SN and MMK:** Participated in conceiving the design of the study, collecting and reviewing the data, and coordinating of project.

**MA, BR and SMT:** Participated in doing literature review, collecting the data and analysis, and preparing the manuscript.

**NI:** Responsible for the accuracy of the study.

All authors approved the final version of the manuscript to be published.
